# Identification of Potential Prognostic Genes for Neuroblastoma

**DOI:** 10.3389/fgene.2018.00589

**Published:** 2018-11-29

**Authors:** Xiaodan Zhong, Yuanning Liu, Haiming Liu, Yutong Zhang, Linyu Wang, Hao Zhang

**Affiliations:** ^1^College of Computer Science and Technology, Jilin University, Changchun, China; ^2^Key Laboratory of Symbolic Computation and Knowledge Engineering, Ministry of Education, Jilin University, Changchun, China; ^3^Department of Pediatric Oncology, The First Hospital of Jilin University, Changchun, China

**Keywords:** neuroblastoma, differentially expressed genes, gene signatures, prognosis, GEO, *ERCC6L*

## Abstract

**Background and Objective:** Neuroblastoma (NB), the most common pediatric solid tumor apart from brain tumor, is associated with dismal long-term survival. The aim of this study was to identify a gene signature to predict the prognosis of NB patients.

**Materials and Methods:** GSE49710 dataset from the Gene Expression Omnibus (GEO) database was downloaded and differentially expressed genes (DEGs) were analyzed using R package “limma” and SPSS software. The gene ontology (GO) and pathway enrichment analysis were established via DAVID database. Random forest (RF) and risk score model were used to pick out the gene signature in predicting the prognosis of NB patients. Simultaneously, the receiving operating characteristic (ROC) and Kaplan-Meier curve were plotted. GSE45480 and GSE16476 datasets were employed to validate the robustness of the gene signature.

**Results:** A total of 131 DEGs were identified, which were mainly enriched in cancer-related pathways. Four genes (*ERCC6L*, *AHCY*, *STK33*, and *NCAN*) were selected as a gene signature, which was included in the top six important features in RF model, to predict the prognosis in NB patients, its area under the curve (AUC) could reach 0.86, and Cox regression analysis revealed that the 4-gene signature was an independent prognostic factor of overall survival and event-free survival. As well as in GSE16476. Additionally, the robustness of discriminating different groups of the 4-gene signature was verified to have a commendable performance in GSE45480 and GSE49710.

**Conclusion:** The present study identified a gene-signature in predicting the prognosis in NB, which may provide novel prognostic markers, and some of the genes may be as treatment targets according to biological experiments in the future.

## Introduction

Neuroblastoma (NB) is a highly heterogeneous pediatric solid tumor both in clinical and biological characteristics. It is the third most common malignant disease, which takes about 7% of malignant tumors in children less than 14 years, with the incidence of close to 60/100,000 during the first year of life (Maris et al., 2007; Sausen et al., 2013; Ward et al., 2014). However, NB has occupied up to 15% of cancer-related death in childhood (Maris et al., 2007; Domingo-Fernandez et al., 2013; Sausen et al., 2013; Salazar et al., 2016; Stafman and Beierle, 2016). Different from adult cancers, NB presents a low frequency of mutation and rearrangement, which only takes up less than 40% (Pugh et al., 2013). Multiple “omics” studies have found that some molecules are involved in NB development, including *MYCN*, *ALK*, *LMO1*, *PHOX2B*, *ARID1A*, and *ARID1B* (Huang and Weiss, 2013; Pugh et al., 2013; Sausen et al., 2013; Beckers et al., 2015; Bosse and Maris, 2016; Liu and Thiele, 2017). Nonetheless, the survival time of the high-risk group has not been markedly prolonged, with the long-term survival of less than 40∼50% (Maris et al., 2007; Pinto et al., 2015; Salazar et al., 2016).

Typically, patients at stage 4 and stage 4s all have metastatic tumors, but they have remarkably different outcomes. Notably, most patients with stage 4 diseases are associated with high risks and inferior outcomes regardless of multi-modal therapy (Maris et al., 2007; Whittle et al., 2017). In contrast, stage 4s diseases frequently occur in infants, and they generally have beneficial outcomes even though without any treatment or moderate chemotherapy (Rubie et al., 2011; Brodeur and Bagatell, 2014). Studies indicate that more than 20% NB cases have experienced spontaneous remission or regression, especially for stage 4s cases (Diede, 2014; Attiyeh and Maris, 2015). In addition, some studies have found the differences between stage 4 and stage 4s patients. For instance, Taggart et al. (2011) had accessed 0–18 months old patients with stage 4 and stage 4s diseases, and found that the tumor biological features [such as *MYCN*, 11q, mitosis-karyorrhexis index (MKI), histology, and 1p] were more important than age and metastatic pattern for predicting the clinical outcomes, which should be considered for risk stratification in patients aged less than 18 months. Moreover, Fischer et al. (2006) had identified a special gene expression pattern between stage 4 and stage 4s patients. Bénard et al. (2008) had identified a stage 4s gene signature by comparing the gene expression profiles between stage 4 and stage 4s patients. However, this is far from enough, and further studies are needed, which may provide some inspiration for understanding and exploiting the therapeutic strategies of NB.

Random forest (RF) is among the most important machine learning methods thanks to their relatively good accuracy, robustness, and ease of use. They can be used to rank the importance of features in a regression or classification problem in a natural way. Mean decrease impurity and mean decrease accuracy are employed as criteria for feature selection (Strobl et al., 2007).

In this paper, we have identified a four-gene signature via RF and risk score model in predicting the prognosis of NB patients via the GEO dataset (GSE49710), the gene signature also had good performance in discriminating other groups of GSE49710, as well as in other two independent datasets. Importantly, the obtained gene signature we picked out could separate NB patients with different outcomes in stage 4 or age less than 18 months, or *MYCN* not amplified. Thus, the gene signature was considered as a prognostic marker of NB, and some genes (like *ERCC6L*) in the signature might serve as the therapeutic targets based on biological experiments in the future.

## Materials and Methods

### Data Collection and Processing

Data were downloaded from GEO datasets GSE49710 (Supplementary Data Sheet S1), GSE45480, and GSE16476. Samples with the survival time of less than 30 days and more than 10 years were excluded. Finally, 419 samples were obtained, and 45 stage 4 patients with the survival time of less than 18 months as well as 50 stage 4s patients were also employed in this study. R package “limma” (Ritchie et al., 2015) was used for data processing, and expression data were transformed by log2 calculation. Meanwhile, the prediction ability was validated using the GSE45480 (GPL16876) and GSE16476 datasets. The highest expression value was employed to represent the gene expression level when a single gene matched multiple probes. Eventually, the top 1% mRNAs with high expression were selected in stage 4 patients and the adjusted *p*-value of < 0.05 was considered as differentially expressed genes (DEGs). Moreover, log-rank test and Cox proportional hazard regression model (Prentice, 1992) were utilized to identify the risk factors related to clinical prognosis. Genes with a hazard ratio of >1 and a *p*-value of less than 0.001 upon univariate Cox regression were picked out.

### Functional Enrichment Analysis of DEGs

Functional enrichment analysis of the candidate DEGs was carried out using the online database DAVID 6.8^1^. In the meantime, gene ontology (GO) (The Gene Ontology Consortium, 2017) term BP (Biological Process) and Kyoto Encyclopedia of Genes and Genomes (KEGG) (Kanehisa et al., 2017) analysis were performed to provide gene biological function, with *p* < 0.05 as the cut-off criterion.

### Selection of Gene Signature and Confirmation of Performance

Firstly, we put the DEGs into the RF model as features, then built the RF model using Scikit-Learn tools. We defined a grid of hyperparameter, and sampled from the grid, performing 10-fold cross-validation with each combination of values. Subsequently, the grid search algorithm outputs the settings that achieved the highest AUC in the validation procedure and the feature importance based on mean decrease impurity (Strobl et al., 2007). Secondly, based on the above ranking results, we combined top 10 important genes to predict the prognosis of patients, then selected one of the optimal combinations, and the coefficient of candidate genes was also calculated with multivariate Cox regression analysis. Thirdly, the risk score model (Zhou et al., 2015; Liao et al., 2018) was adopted to evaluate the performance in predicting the vital status and assess its discrimination ability in other different groups. In addition, the receiving operating characteristic (ROC) curve was plotted.

$$\mathrm{Risk Score}=\sum_{i=1}^{n}\beta_{i}^{*}\chi_{i}$$

where β_i_ indicated the coefficient of each gene in multivariate Cox regression analysis with overall survival as the dependent parameter and χ_i_ represented the expression value by log2 transformation of each gene.

Simultaneously, all samples were divided into the high or low-risk group according to the median risk score, and the Kaplan–Meier curves of overall survival and event-free survival were plotted.

### Statistical Analysis

All statistical analyzes were carried out using the IBM SPSS version 23 software and R. Bivariate coefficient was calculated through Spearman’s rank correlation. Additionally, Kaplan-Meier method and log-rank test were employed to plot and compare the survival curves. Survival data were assessed through univariate and multivariate Cox regression analyses. A two-tailed *p*-value of < 0.05 was considered statistically significant.

## Results

### Clinical Characteristics Analysis of GEO Data

A total of 498 NB samples with complete clinical data were downloaded from the GSE49710 dataset, and 419 of them were finally included in our study. The details of clinical/pathological features were listed in Table 1. Survival analysis showed that INSS stage 4 patients had inferior outcomes to those of stage 4s patients (Supplementary Figure S1). Stage 4 patients had taken up 63.6, 70.0, 77.7, and 83.9% among all the 419 patients with progression, *MYCN* amplification, death from disease and high risk, respectively. In contrast, stage 4s patients with *MYCN* amplification, high risk and death from disease had occupied less than 4.5%, and those with progression had accounted for 7.5%. In comparison, in the TARGET-NBL dataset, stage 4 patients had taken up more than 80% in death, event, progression and relapse groups, and the proportions were especially high in death and relapse groups (96.9, and 91.3%, respectively). However, stage 4s patients had only occupied no more than 2% (Supplementary Table S1). These results demonstrated huge differences between stage 4 and stage 4s patients. Cox regression analysis of stage 4 patients (GSE49710, *n* = 168) revealed that the age of ≥ 18 months, *MYCN* amplification, and high risk were associated with inferior outcomes [hazard ratio: 2.237 (95% CI: 1.209–4.141), 2.437 (95% CI: 1.557–3.814), 11.208 (95% CI: 2.746–45.743) and *p*-value: 0.01, < 0.001, 0.001, respectively]. Results of risk factor analysis in this dataset were consistent with those from related articles (Pinto et al., 2015).

**Table 1:** Clinical characteristics of NB patients in GSE49710 (samples with survival time ≤30 days and ≥10 years have been removed).

Characteristics	Number of cases (%)
**Gender**	
Male	247 (58.9)
Female	172 (41.1)
**Age at Diagnosis**	
<18 months	246 (58.7)
≥18 months	173 (41.3)
**MYCN Status**	
Amplified	90 (21.5)
Not Amplified	324 (77.3)
N/A	5 (1.2)
**Risk Group**	
High risk	168 (40.1)
Intermediate or low risk	251 (59.9)
**INSS Stage**	
1	90 (21.5)
2	65 (15.5)
3	46 (11.0)
4	168 (40.1)
4s	50 (11.9)
**Class Label**	
Favorable	141 (33.7)
Unfavorable	90 (21.5)
N/A	188 (44.9)
**Progression**	
Yes	173 (41.3)
No	246 (58.7)
**Death from Disease**	
Yes	103 (24.6)
No	316 (75.4)

*N/A, not applicable; Class label, Maximally divergent disease courses; unfavorable, patients died despite intensive chemotherapy; favorable, patients survived without chemotherapy for at least 1000 days post-diagnosis.*

### Identification of DEGs and Functional Enrichment Analysis in NB

In this study, 45 INSS stage 4 patients aged less than 18 months and 50 stage 4s patients were enrolled from the GSE49710 dataset, so as to exclude the influence of age. According to the cut-off criterion of adjusted *p*-value of < 0.05, the top 1% high expression genes of 26082 mRNAs in stage 4 patients were selected, and a total of 215 differentially expressed mRNAs were identified after excluding the repetitive genes. Afterward, genes with the hazard ratio of > 1 and *p*-value of < 0.001 upon univariate Cox regression were chosen, and 131 DEGs were selected at last (Supplementary Table S2).

**FIGURE 1:**
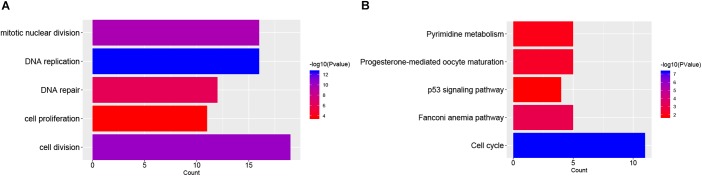
Functional enrichment analysis of DEGs in NB. **(A)** The top 5 gene count of gene ontology (GO) terms in NB-related genes; **(B)** The top 5 significant KEGG pathways in NB-related genes.

The functions and pathway enrichment of the candidate DEGs were analyzed using online database DAVID, and 58 GO terms in BP (Biological Process) and 5 KEGG pathways were revealed finally (Figures 1A,B and Supplementary Tables S3, S4). As shown in Figure 1A, the DEGs were mainly enriched in cell division, DNA replication, mitotic nuclear division, DNA repair and cell proliferation (Stafman and Beierle, 2016). In terms of KEGG pathways, the DEGs were mainly enriched in cell cycle (Williams and Stoeber, 2012; Otto and Sicinski, 2017), Fanconi anemia pathway (Ceccaldi et al., 2016), pyrimidine metabolism (Kelemen et al., 2014), and p53 signaling pathways, which were the cancer-related pathways.

### Performance of Gene Signature in Predicting Prognosis

**FIGURE 2:**
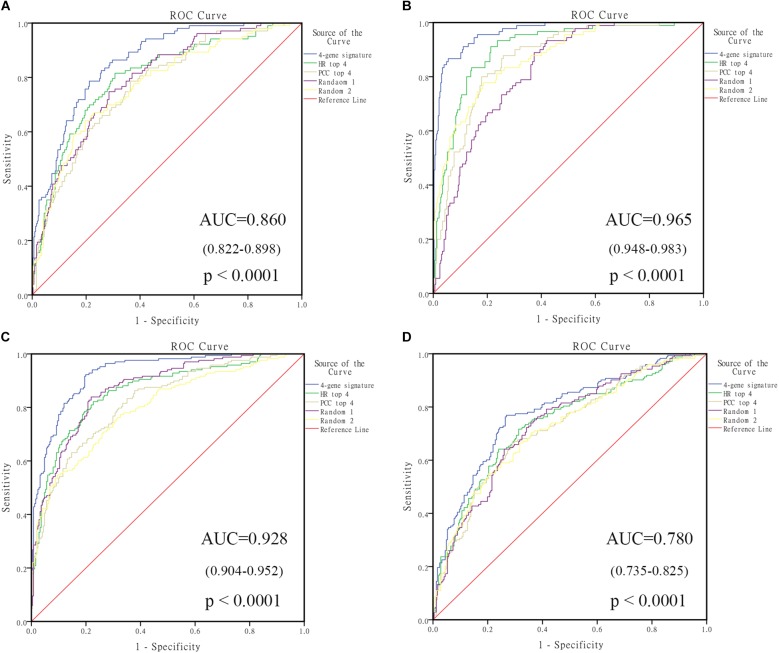
The receiving operating characteristic curve (ROC) of GSE49710. **(A)** ROC of vital status; **(B)** ROC of MYCN amplified; **(C)** ROC of high risk; **(D)** ROC of progression. HR, hazard ratio; PCC, Pearson correlation coefficient.

In order to select the genes with good performance in predicting prognosis of NB, RF was adopted to rank the 131 DEGs in predicting the prognosis in NB patients (Supplementary Table S5). Based on the ranking results, four genes (*ERCC6L*, *AHCY*, *STK33*, and *NCAN*) were selected as a gene signature in predicting the vital status of NB patients (Table 2). To integrate four genes, multivariate Cox regression analysis was employed to obtain the coefficient. The risk score was calculated as follows, risk score = expression of *ERCC6L*^∗^0.408 + expression of *AHCY*^∗^0.478 + expression of *STK33*^∗^0.345 + expression of *NCAN*^∗^0.136. Notably, the AUC of the 4-gene signature in predicting vital status could reach 0.86. Simultaneously, the 4-gene signature was employed to distinguish different groups, including *MYCN* amplification vs. Non-amplification, high risk vs. non-high risk, and progression vs. Non-progression. According to the ROC curve, the AUC of *MYCN* amplification, high risk, and progression reached 0.965, 0.928, and 0.78, respectively, as shown in Figure 2. Surprisingly, *AHCY* performed excellently in predicting *MYCN* amplification, with an AUC of 0.946 (Supplementary Table S6).

**Table 2:** Top 10 genes with high Gini importance by the random forest algorithm.

Accession no.	Gene symbol	Gini importance	logFC	adj.P.Value	Chr	HR (OS)	95%CI	*P-*value
NM_004386	**NCAN**	**0.061850062**	1.697	0.002175968	chr19	1.475	1.345–1.617	<0.0001
NM_000687	**AHCY**	**0.038477178**	0.686	0.000319358	chr20	3.131	2.494–3.932	<0.0001
NM_030906	**STK33**	**0.034351926**	1.202	0.001117677	chr11	1.798	1.569–2.061	<0.0001
NM_014501	UBE2S	0.028596074	0.84	3.01E-05	chr19	2.943	2.355–3.679	<0.0001
NM_003686	EXO1	0.021770529	0.953	0.001977921	chr1	2.148	1.811–2.548	<0.0001
NM_017669	**ERCC6L**	**0.020307985**	0.952	0.000570824	chrX	2.294	1.920–2.741	<0.0001
NM_016354	SLCO4A1	0.018810664	1.747	0.001106007	chr20	1.463	1.341–1.597	<0.0001
NM_001827	CKS2	0.018428233	0.678	0.004725484	chr9	2.679	2.179–3.294	<0.0001
NM_002358	MAD2L1	0.018307845	0.777	0.007867488	chr4	2.082	1.744–2.486	<0.0001
NM_032117	MND1	0.018163659	0.849	0.002682907	chr4	2.381	1.985–2.885	<0.0001

*Bold values indicate the genes we selected as the signature.*

### Association of 4-gene Signature and Clinical Characteristics

**FIGURE 3:**
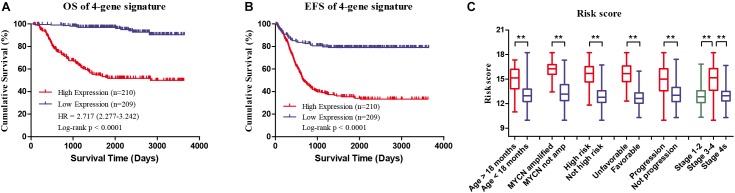
Clinical Features of high risk score group and low risk score group of the four-gene signature in GSE49710. **(A)** Overall survival of high/low risk score group. **(B)** Event-free survival of high/low risk score group. **(C)** Risk score in different groups, ^∗∗^*p* < 0.0001.

**FIGURE 4:**
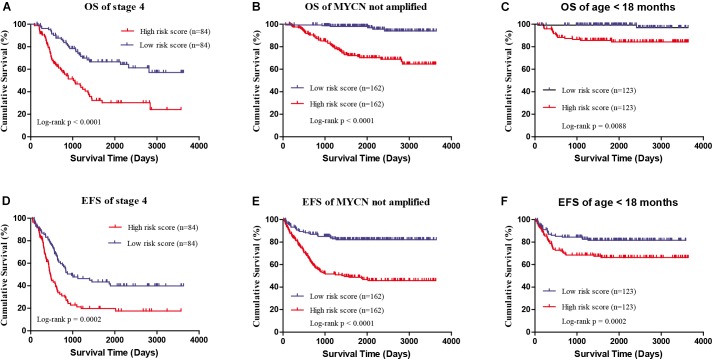
The survival analysis of high/low risk score group of the four gene signature with exclusion of known risk factors. **(A,D)** OS and EFS in stage 4 patients. **(B,E)** OS and EFS of MYCN not amplified patients. **(C,F)** OS and EFS of age less than 18 months patients.

Four genes (*ERCC6L*, *AHCY*, *STK33*, and *NCAN*) were closely correlated with overall survival and event-free survival. As shown in Supplementary Figures S2A–H, the increased expression of the four genes was linked with markedly shorter survival time. In risk score model, the group with high risk score of four gene-signature had significant inferior outcomes in NB patients (Figures 3A,B). Meanwhile, the risk score in different groups was calculated, and the results revealed that a high age, *MYCN* amplification, advanced stage, high risk, disease progression, and unfavorable class label groups had high risk scores (*p* < 0.0001) (Figure 3C). Moreover, the overall survival and event-free survival were further assessed in stage 4 patients, as well as those with *MYCN* non-amplification, and age of less than 18 months with high/low risk score. The results showed that the high-risk group had significant short overall and event-free survival time (Figure 4).

Spearman analysis of the correlation between 4-gene signature and clinicopathological features revealed that the high risk score group showed markedly positive correlation with age, risk, and INSS stage, while negative correlation with overall survival and event-free survival time. In addition, the high risk score group was also associated with unfavorable class label and *MYCN* amplification, as summarized in Table 3. Taken together, these known risk factors of the prognosis of NB (Pinto et al., 2015) were consistent with the 4-gene signature-based risk score selected in this study.

**Table 3:** Spearman analysis of correlation between four-gene signature and clinicopathological features in GSE49710.

Variables	Risk score group	
	spearman correlation	*P*-value
Age at diagnosis	0.530	<0.0001
MYCN amplified	0.577	<0.0001
High Risk	0.714	<0.0001
INSS stage	0.588	<0.0001
Class label	0.767	<0.0001
Progression	0.473	<0.0001
Death from disease	0.533	<0.0001
EFS days	-0.367	<0.0001
OS days	-0.362	<0.0001

Univariate analysis suggested that the 4-gene signature, age group (age of ≥ 18 months and age of < 18 months), *MYCN* amplification, high risk, and INSS stage were the markedly prognostic factors of the overall survival and event-free survival in NB (all *p*s < 0.0001) (Table 4). Besides, multivariate survival analysis was also performed using the variables with significance in univariate analysis. The results confirmed that the 4-gene signature and INSS stage remained independent prognostic indicators for unfavorable overall survival and event-free survival (Table 4).

**Table 4:** Univariate and multivariate Cox regression analysis of the correlation between the 4-gene signature and clinical features in GSE49710.

	Univariate analysis			Multivariate analysis		
Variables	*P*-value	Hazard ratio	95% CI	*P*-value	Hazard ratio	95% CI
4-gene signature	<0.0001	1.095	1.079–1.112	**<0.0001**	1.958	1.530–2.507
Age group	<0.0001	7.634	4.678–12.458	0.098	1.716	0.904–3.256
MYCN amplified	<0.0001	6.001	4.048–8.896	0.166	1.386	0.874–2.198
High risk	<0.0001	16.643	9.274–29.868	0.323	1.666	0.606–4.583
INSS stage	<0.0001	3.123	2.300–4.241	**0.023**	1.563	1.063–2.297

*Overall survival. Bold values emphasize p < 0.05.*

### Confirmation of Potential Function of ERCC6L in NB

**FIGURE 5:**
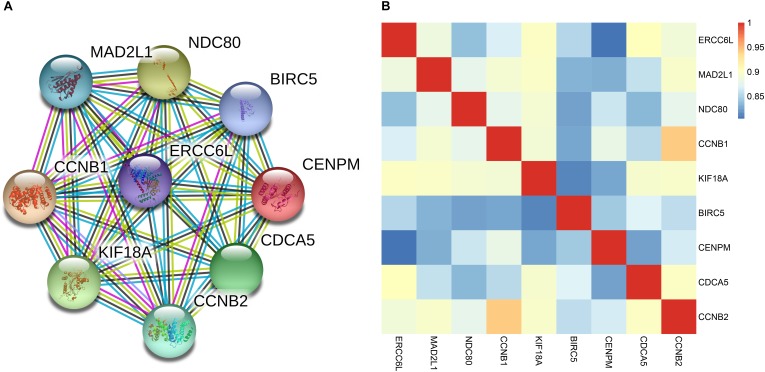
Correlation of ERCC6L with other key genes in NB. **(A)** The protein-protein interaction network of ERCC6L and other key genes; **(B)** Pearson correlation coefficient of 9 genes in NB.

In the four-gene signature, the AUC of *ERCC6L* reached 0.799 in predicting the survival status of NB patients, which was the highest. In order to obtain the potential function of *ERCC6L* in NB development, the gene-encoded protein–protein interaction (PPI) network was constructed by employing the STRING database^2^, so as to find the co-expression relationship of genes. CytoHubba (Chin et al., 2014) and MCODE (Bader and Hogue, 2003) of Cytoscape (Shannon et al., 2003) software were employed to screen the hub genes that interacted with ERCC6L, and 8 genes (including *MAD2L1*, *NDC80*, *CCNB1*, *KIF18A*, *BIRC5*, *CENPM*, *CDCA5*, and *CCNB2*) with the Clustering Coefficient of > 0.9 were selected, as shown in Figure 5A. And the Pearson correlation coefficient (PCC) of the nine genes was also calculated. As shown in Figure 5B, the PCC of every two genes was higher than 0.8 (*p*-value < 2.2E-16) (PCC of all DEGs in Supplementary Table S8). The above results suggested that *ERCC6L* might interact with one or more genes in the development of NB. Nevertheless, the mechanism of *ERCC6L* in NB should be further confirmed from biological experiments.

### Validation of Performance in Other NB Datasets

**FIGURE 6:**
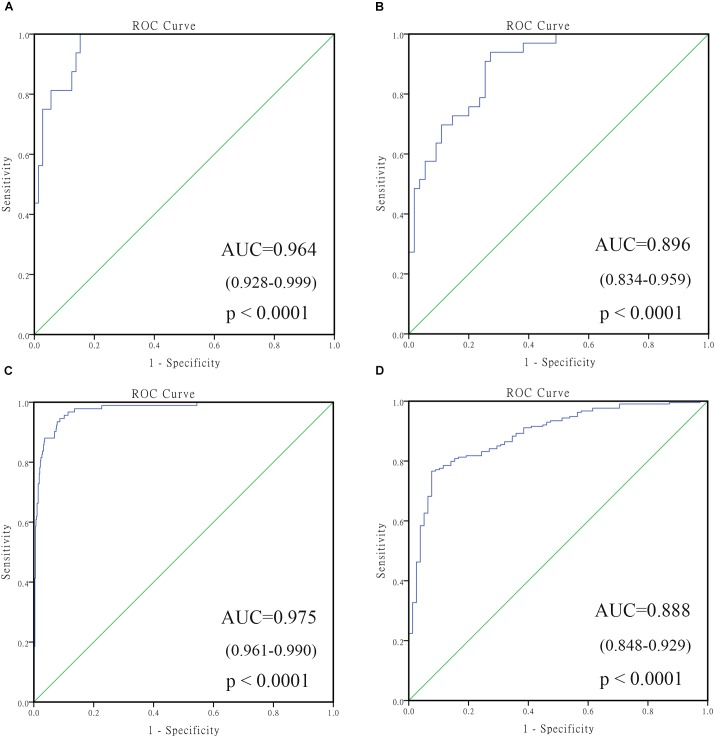
The receiving operating characteristic curve (ROC) of validated datasets. **(A)** ROC of MYCN status in GSE16476; **(B)** ROC of vital status in GSE16476; **(C)** ROC of MYCN status in GSE45480; **(D)** ROC of stage 4 vs. stage 4s in GSE45480.

To confirm the robustness of the 4-gene signature in predicting different groups, two independent datasets GSE16476 and GSE45480 (GPL16876) were used for evaluation. As shown in Figure 6, the 4-gene signature had a commendable performance in dividing *MYCN* amplification and non-amplification groups, with the AUC of 0.964 and 0.975, respectively. Meanwhile, the AUC of the 4-gene signature was 0.896 and 0.888, respectively, in the vital status prediction of GSE16476 and discrimination of stage 4 and stage 4s in GSE45480. Through analyzing the association of 4-gene signature with clinical features in GSE16476, we obtained similar results as GSE49710 (Supplementary Figures S3, S4 and Supplementary Table S7). We divided patients into high/low risk score group based on the median of 4-gene signature, the results showed that high risk score group correlated with age more than 18 months, advanced stage, MYCN amplified, and disease recurrence or progression, and had negative correlation with follow-up time.

## Discussion

NB, an embryonal malignant tumor during early childhood, will lead to poor long-term survival, especially for stage 4 patients. Stratified treatment has achieved great progress in the past two decades (Pinto et al., 2015; Whittle et al., 2017); in the meanwhile, *MYCN* amplification and *ALK* mutation have been comprehensively studied as the prognostic factors and important treatment targets (Barone et al., 2013; Huang and Weiss, 2013; Beckers et al., 2015; Liu and Thiele, 2017). However, *MYCN* amplification accounts for only about 25% patients (Huang and Weiss, 2013). Consequently, identifying key genes, exploring new targets for therapy, and ultimately improving survival time of patients with inferior outcomes remain challenging.

This study had focused on discriminating NB with tremendously different clinicopathological features, identifying the differentially expressed genes (DEGs) in the untreated stage 4 and stage 4s patients, and utilizing bioinformatic means to intensively analyze the data. We had identified 131 DEGs, and BP in GO term and KEGG functional enrichment analysis showed that the DEGs were mainly enriched in the cancer-related pathways, such as DNA replication, cell division and cell cycle (Williams and Stoeber, 2012; Otto and Sicinski, 2017). We picked out a four-gene (*ERCC6L*, *AHCY*, *STK33*, and *NCAN*) signature in predicting the prognosis of NB, with the AUC of as high as 0.86. Simultaneously, the grouping ability of the gene signature was assessed, the results of which revealed that the four-gene signature had good performance in predicting other groups. When predicting the *MYCN* amplification group, the AUC of the four-gene signature was 0.965, and in another two independent datasets, the AUC in predicting *MYCN* amplification was higher than 0.95. We guessed there are two reasons: (1) the AUC in predicting *MYCN* amplification of *AHCY* alone was 0.946, which could be attributed to the fact that *AHCY* was directly regulated by *MYC* proteins (Chayka et al., 2015); (2) might be due to the insufficient sample size and imbalanced distribution of positive and negative samples.

An ideal prediction model should help clinicians to determine optimal treatment strategies or aid them to predict patient outcomes. However, it is difficult to develop a satisfactory prognostic model, which can be ascribed to extreme cancer complexity. *MYCN* amplification, *ALK* mutation (Barone et al., 2013; Lambertz et al., 2015), or other molecular biomarkers (Egler et al., 2008; Pugh et al., 2013; Zage et al., 2013) have provided researchers with some hints, yet more studies are still needed. In particular, current researches mainly focused on how to reduce the treatment-associated side effect in patients with good prognosis, and how to provide more effective treatment for patients with poor prognosis patients (Whittle et al., 2017). This requires researchers to find more markers that distinguish patients with good prognosis and poor prognosis. Our study identified a four-gene signature in predicting prognosis and discriminating other groups, which had attained satisfactory performance.

Multivariate Cox regression analysis showed that the four-gene signature and INSS stage are all independent prognostic indicators. The two features are highly correlated and have a prognostic significance, this indicated that there is a part of coincident information between them, but the signature also has its own independent prognostic information, and it contributes more to the prognosis analysis of NB patients according to the *p*-value. In addition, in order to support our results, we removed the INSS stage and applied other features to analyze again. The results showed that the signature was still an independent prognostic indicator, further validating the effect of our signature. At the same time, survival analysis of the four-gene signature in stage 4 and *MYCN* non-amplification patients, and age of < 18 months was also performed, and the results indicated that the high risk score group had significantly inferior outcomes. In conclusion, our four-gene signature might help clinicians to choose the treatment regimens and aid them to predict patient prognosis in a similar situation. We got similar results in the independent dataset GSE16476, which can support the robustness of our signature in predicting the prognosis in NB patients.

Of the four genes, *AHCY* has been reported to be a direct target of *MYCN*, which predicts poor prognosis in NB (Chayka et al., 2015). NCAN has been confirmed to promote the malignant phenotypes in NB cells (Su et al., 2017). ERCC Excision Repair 6 Like (*ERCC6L*, Spindle Assembly Checkpoint Helicase) is a protein-coding gene, also known as PICH, its related pathways include cell cycle and mitosis. Pu et al. had found that higher *ERCC6L* expression was notably associated with inferior outcomes in breast and kidney cancers, and silencing of *ERCC6L* would inhibit the growth of breast and kidney cancer cells. Besides, their further studies considered that *ERCC6L* might affect the cell cycle via RAB31-MAPF-CDK, so as to promote cancer cell proliferation (Pu et al., 2017). Albers et al. showed that loss of PICH results in DNA damage, P53 activation, and embryonic development impaired (Albers et al., 2018), which indicated that PICH may play an important role in embryonic lethality and tumorigenesis. In our study, univariate and multivariate Cox regression analyses revealed that *ERCC6L* could be an independent prognostic factor of overall survival and event-free survival (*p* < 0.0001). Typically, the AUC of *ERCC6L* alone in predicting different groups was higher than 0.75 (*p* < 0.0001).

In order to find the potential function of *ERCC6L*, we have constructed a PPI network and calculated the PCC of *ERCC6L* with other key genes which may interact with *ERCC6L*, eight genes had been identified that had a close relationship with it. Most of them were involved in the cell cycle pathway. Importantly, of the eight genes, *MAD2L* (Gogolin et al., 2013), *CCNB1* (Liu et al., 2013; Schwermer et al., 2015), and *BIRC5* (Lamers et al., 2011; Hagenbuchner et al., 2016) have been proven play important roles in NB development and metastasis. Taken together, we considered *ERCC6L* may play an important role in NB, biological experiments are needed for further study of its mechanism.

## Author Contributions

YL conceived of and directed the project. XZ designed the study, analyzed the data, wrote the manuscript. HL revised the manuscript critically for important intellectual content. YZ collected data and samples. LW reviewed the data. All authors have read and approved the final manuscript for publication.

## Conflict of Interest Statement

The authors declare that the research was conducted in the absence of any commercial or financial relationships that could be construed as a potential conflict of interest.

## Footnotes

1 https://david.ncifcrf.gov/

2 https://string-db.org/
