# Supplementary figures and images for: Identification of Potential Prognostic Genes for Neuroblastoma
Source: Front Genet. 2018 Nov 29;9:589. doi: 10.3389/fgene.2018.00589 (PMC6282001; doi:10.3389/fgene.2018.00589)

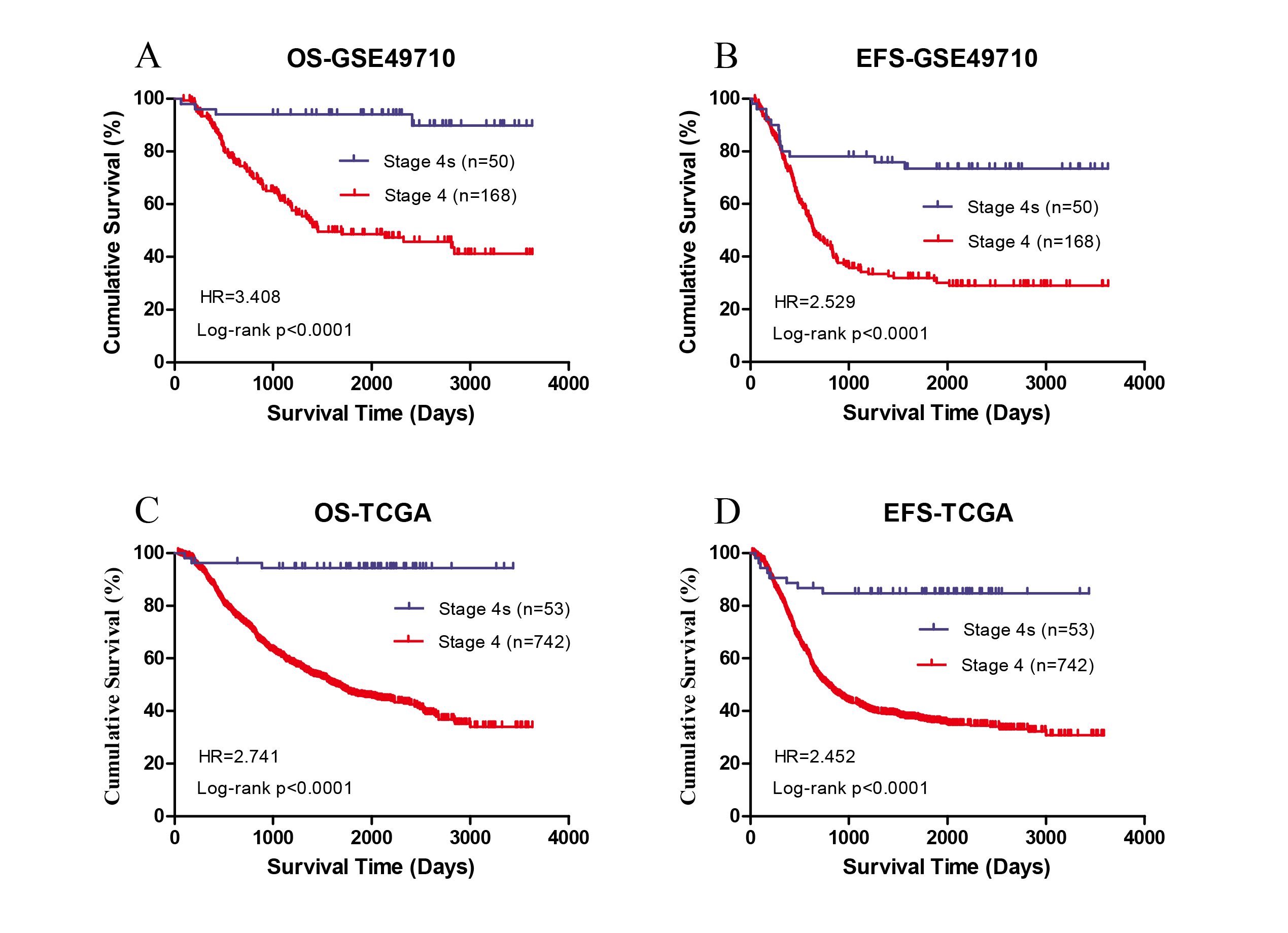

Supplement: Supplementary file 1 [file Image_1.TIF]

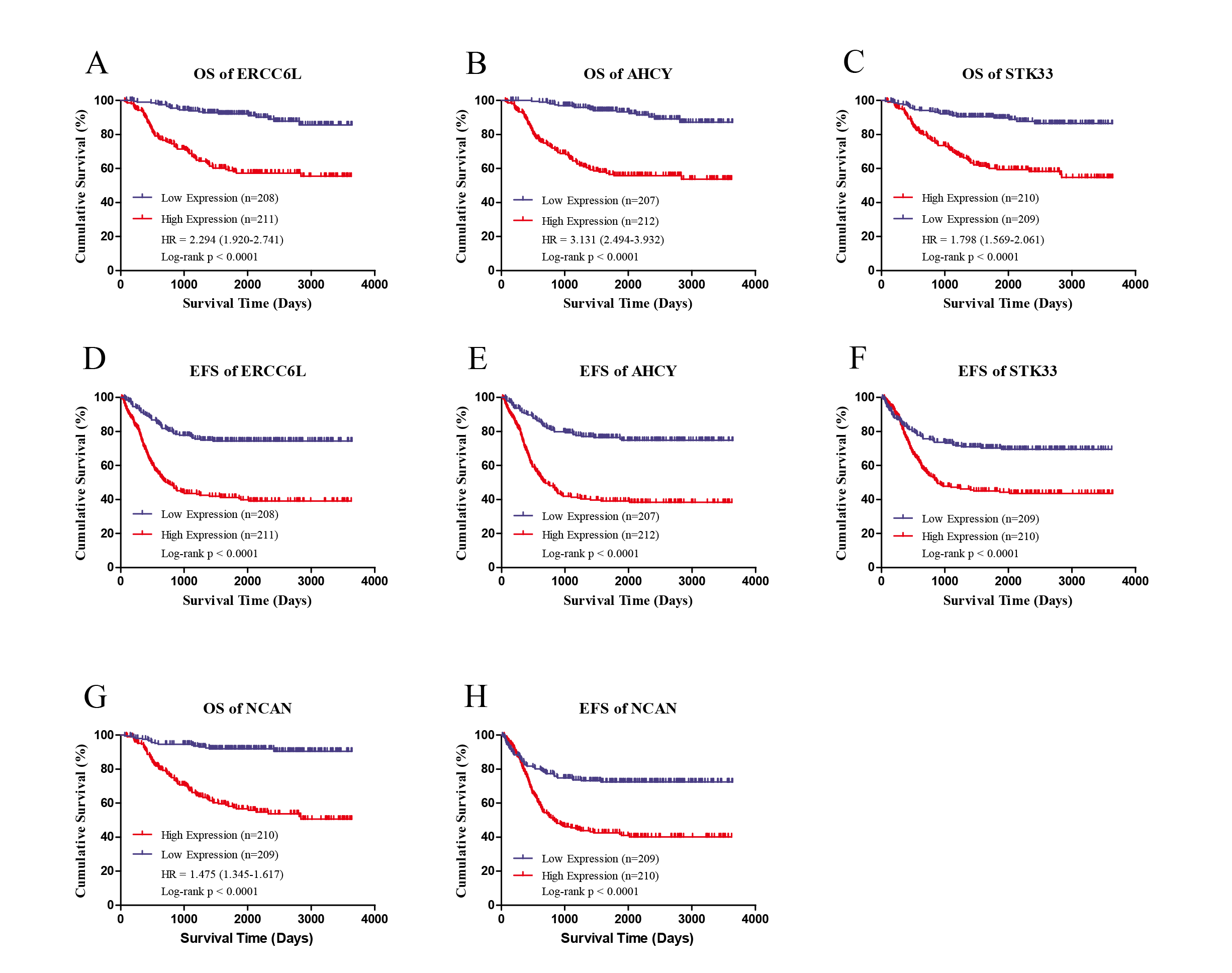

Supplement: Supplementary file 2 [file Image_2.TIF]

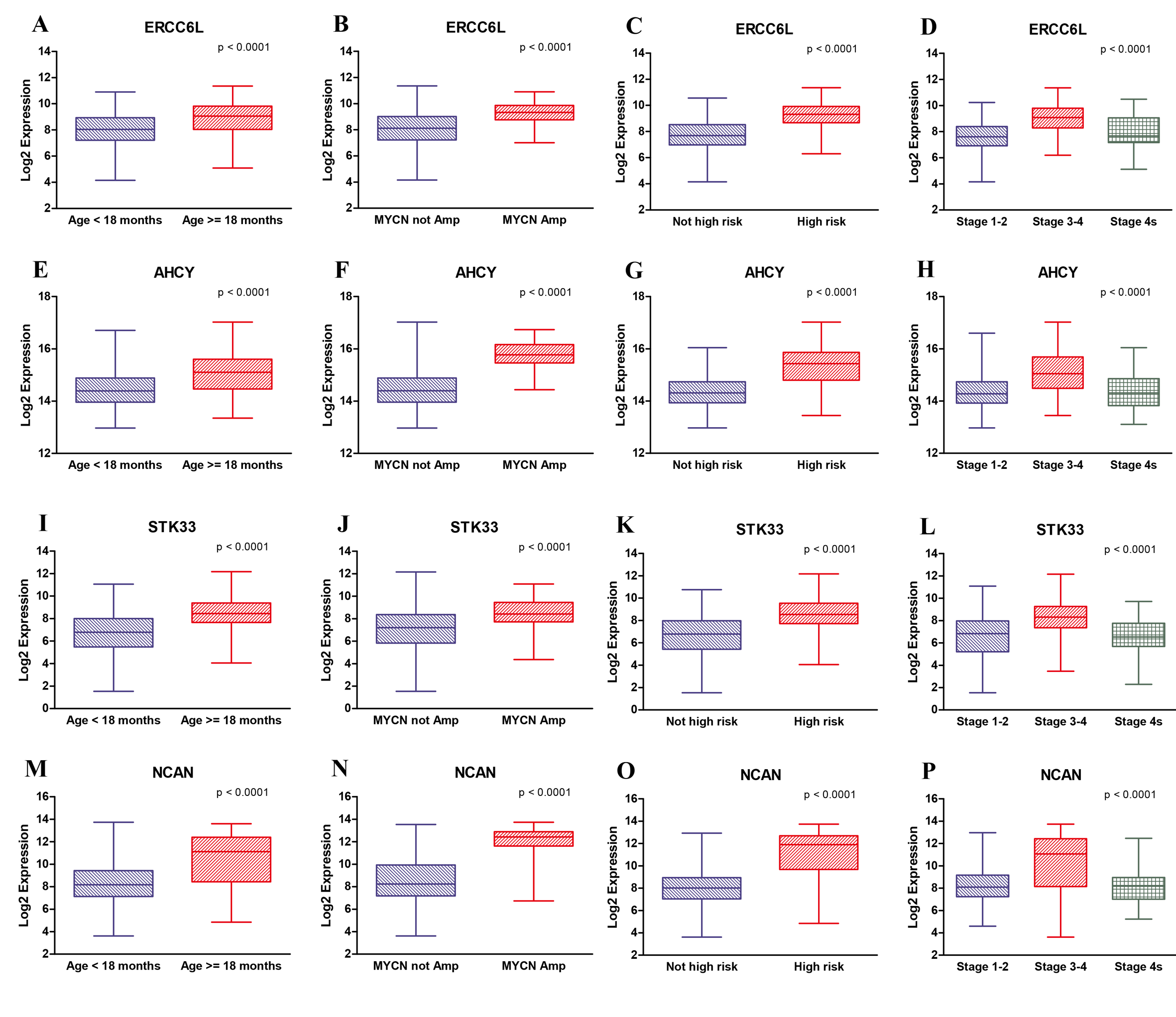

Supplement: Supplementary file 3 [file Image_3.TIF]

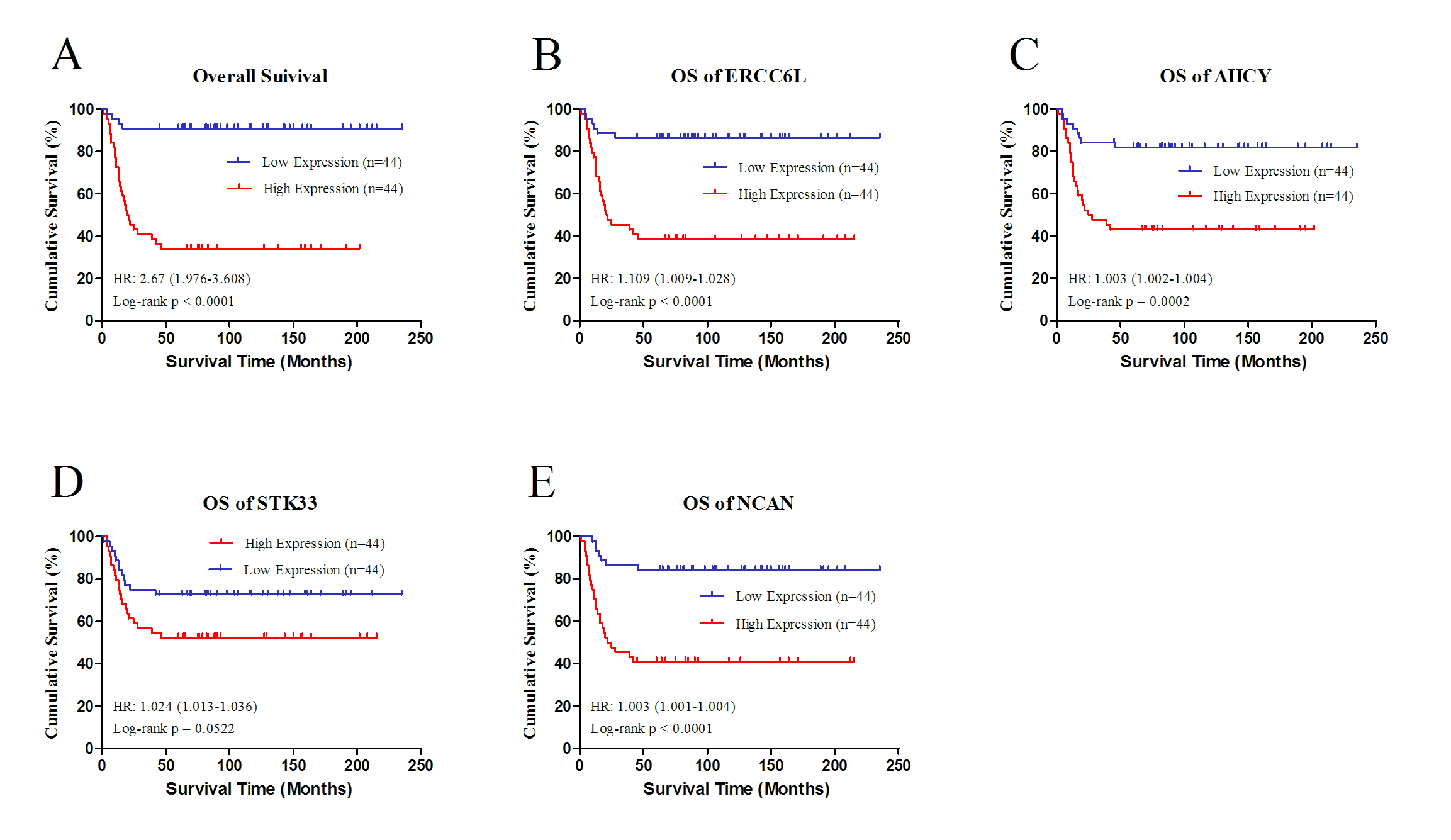

Supplement: Supplementary file 4 [file Image_4.TIF]
