# Supplementary material for: Identification of Potential Prognostic Genes for Neuroblastoma
Source: Front Genet. 2018 Nov 29;9:589. doi: 10.3389/fgene.2018.00589 (PMC6282001; doi:10.3389/fgene.2018.00589)
Supplement: Supplementary file 7 [file Data_Sheet_2.doc]

**Figure S1**. Kaplan-Meier curve of stage 4 vs. stage 4s patients in GSE49710 and TCGA. HR: hazard ratio.

**Figure S2**. Correlation of 4 genes with clinical features. (A)-(H) Kaplan-Meier curves of four prognostic mRNAs in NB.

** denotes p < 0.0001. Abbreviations: OS, overall survival; EFS, event-free survival.

**Figure S3**. Boxplot of 4 genes in different groups in GSE49710.

**Figure S4.** OS of four-gene signature and 4 genes in GSE16476. (A) OS of four-gene signature in high/low risk score group; (B)-(E) OS of ERCC6L, AHCY, STK33 and NCAN with high/low expression.
